# Ehrlichiosis-Associated Hemophagocytic Lymphohistiocytosis: A Case Series and Review of the Literature

**DOI:** 10.1155/2023/5521274

**Published:** 2023-02-15

**Authors:** Kassem Hammoud, Robert Fulmer, Megan Hamner, Wissam El Atrouni

**Affiliations:** ^1^Department of Infectious Diseases, University of Kansas Medical Center, Kansas City, Kansas, USA; ^2^Department of Infectious Diseases, Spectrum Health, Grand Rapids, Michigan, USA; ^3^Department of Pediatric Infectious Diseases, University of Mississippi Medical Center, Jackson, Mississippi, USA

## Abstract

**Background:**

Human monocytic ehrlichiosis (HME) is a potentially life-threatening tick-borne illness. HME-associated hemophagocytic lymphohistiocytosis (HLH) is a rare entity with a paucity of published literature regarding treatment and outcome. We present the clinical features, treatment, and outcomes of 4 patients at our institutions with HME-associated HLH. This review also summarizes the current literature regarding the presentation, treatment, and outcome of this infection-related HLH.

**Methods:**

We searched the PubMed database for case reports and case series. All cases were diagnosed according to the HLH-04 criteria.

**Results:**

Four cases of HME-associated HLH were included from our institutions. The literature review yielded 30 additional cases. About 41% of the cases were in the pediatric population; 59% were female; and all patients had fever, cytopenia, and elevated ferritin. Most patients were immunocompetent; all but one patient with available data were treated with doxycycline, and eight of the patients with available data received the HLH-94 treatment protocol. The mortality rate was 17.6%.

**Conclusions:**

HME-associated HLH is a rare but serious syndrome with significant mortality. Early treatment with doxycycline is critical, but the role of immunosuppressive therapy is individualized.

## 1. Introduction

Human monocytic ehrlichiosis (HME) is a tick-borne illness caused by *Ehrlichia chaffeensis* in the southeast and southcentral United States, where the vector is the *Amblyomma americanum* (Lone star) tick [1]. Hemophagocytic lymphohistiocytosis (HLH) is an increasingly recognized and often life-threatening syndrome of excessive immune activation. Primary HLH is triggered by genetic disorders and usually manifests in children under the age of 18 months. Secondary HLH is triggered by numerous conditions, like infection or alterations in immune homeostasis as with malignancies, rheumatologic conditions, or immunodeficiency syndromes [2].

The most common infectious trigger is a viral infection like Epstein-Barr virus, but many other microorganisms like fungal or bacterial pathogens can be implicated [3]. The diagnosis is usually guided by the HLH-04 criteria [4]. HLH-04 criteria require five of the following: (1) fever; (2) splenomegaly; (3) cytopenia in two or more blood cell lines; (4) hypertriglyceridemia (>265 mg/dL) or hypofibrinogenemia (<150 mg/dL); (5) hemophagocytosis in the bone marrow, spleen, liver, or lymph nodes; (6) hyperferritinemia (>500 ng/mL); (7) impaired NK cell function; and (8) elevated soluble CD25 (soluble IL-2 receptor alpha) two standard deviations above age-adjusted laboratory-specific norms. HLH may present as a single episode of disease or as recurrent episodes.

The treatment of secondary HLH is most effective when the inciting disease can be treated and controlled. If patients fail to improve or deteriorate, they may benefit from treatment with regimens such as the HLH-94 protocol (dexamethasone and etoposide) [5]. HME-associated HLH is a rare disorder with limited published data regarding treatment. We describe the clinical features, treatment, and outcomes of 4 local cases. In addition, we review the current literature about the diagnosis, clinical and laboratory manifestations, and treatment of HME-associated HLH.

## 2. Literature Review

### 2.1. Methods

We searched the PubMed database for “ehrlichiosis” and “hemophagocytic syndrome,” “Ehrlichia induced hemophagocytic syndrome,” “HLH” and “ehrlichiosis,” and “Ehrlichia associated HLH.” We included all case reports and case series with a published abstract in English. A medical records search of adult patients with HLH seen at our institution from 1/1/2006 to 9/30/2017 yielded only 2 cases of HME-associated HLH; the third case was seen in 2021.

## 3. Results

### 3.1. Case Series

We included three cases seen at The University of Kansas Medical Center in Kansas City, Kansas, and one case seen at Children's Mercy Kansas City Hospital in Kansas City, Missouri. All four patients met the HLH-04 criteria for diagnosis.

**Case 1 .**

A previously healthy 4-year-old female was admitted with 4 days of headache and fever up to 104 F (40 C). Three days prior to admission, she developed myalgia, diarrhea, and a petechial rash on the face, trunk, and extremities. She recently travelled with her family to a lake house in Missouri. On admission, she had a petechial rash and was ill-appearing and fussy. Her laboratory tests showed white blood cells (WBC) 2.84 K/uL, platelets 33 K/uL, transaminitis, ferritin > 10000 ng/mL, fibrinogen < 60 mg/dL, soluble IL-2 receptor 10,364 pg/mL (normal: 175–858), *Ehrlichia chaffeensis* IgG was elevated at 1 : 128 and the blood polymerase chain reaction (PCR) was positive for *Ehrlichia chaffeensis*. She was started on steroids and treated with a 10-day course of doxycycline (started on admission), eventually recovering and being discharged home.

**Case 2 .**

A 67-year-old female from rural Missouri with a history of autoimmune hepatitis and cirrhosis, maintained on azathioprine, was hospitalized with fever, altered mental status, myalgias, weakness, and fatigue of one week duration. Her laboratory tests showed WBC 0.4 K/uL, platelets 7 K/uL, transaminitis, ferritin > 7500 ng/mL, LDH 3415 U/L, fibrinogen 93 mg/dL, and soluble IL2 receptor 2916 pg/mL. She was diagnosed with septic shock and met the criteria for HLH (Table 1). A broad infectious workup was pursued. *Ehrlichia chaffeensis* PCR was positive. Blood PCRs for Epstein–Barr virus (EBV), Cytomegalovirus (CMV), and Primate Erythroparvovirus 1 (previously Parvovirus B19) were also detected, but were quantitatively very low and not thought to be contributing to her illness. Doxycycline was started promptly on admission, and dexamethasone was added later, when suspicion for HLH became high. A few weeks after admission, she developed a relapse of severe thrombocytopenia that promptly responded to reinitiation of doxycycline with subsequent improvement and discharge home.

**Case 3 .**

A 69-year-old female with alcohol use disorder was admitted with confusion, fever, diarrhea, and thrombocytopenia. She developed a seizure and required endotracheal intubation and mechanical ventilation. Her laboratory tests showed WBC 3.6 K/uL, platelets 20 K/uL, hemoglobin 7.5 g/dL, ferritin > 7500 ng/mL, fibrinogen 135 mg/dL, triglycerides 504 mg/dL, soluble IL2 receptor 12,540 pg/mL, transaminitis, bone marrow biopsy with increased histiocytes with hemophagocytosis, and blood *Ehrlichia chaffeensis* PCR was positive. She had a negative infectious workup, including HIV antigen/antibody, blood PCR for EBV, CMV, and Primate Erythroparvovirus 1, an acute hepatitis panel, and Rocky Mountain spotted fever serology. Cerebrospinal fluid analysis and culture did not show evidence of meningitis. She also developed myocarditis, likely related to HME, with serum troponin I > 70 ng/mL. She was diagnosed with HLH and was treated with doxycycline (started 3 days after the initial hospitalization) and the HLH-94 protocol (dexamethasone and etoposide). Her hospital stay was complicated by bacterial pneumonia and severe renal failure. She slowly improved and was discharged home. On outpatient follow-up 3 months later, she was continuing to improve.

**Case 4 .**

A 47-year-old man, previously healthy, was transferred to our institution from a rural hospital with altered mental status, progressive generalized weakness, tachycardia, tachypnea, and fever. The initial workup revealed multisystem organ failure with transaminitis, acute renal failure (creatinine 11.0 mg/dL), leukopenia (3.1 K/uL), thrombocytopenia (22 K/uL), and fibrinogen of 155 mg/dL. He was started on broad-spectrum antimicrobials, including doxycycline; hemodialysis was initiated; and he required mechanical ventilation. His cerebrospinal fluid (CSF) analysis showed neutrophilic pleocytosis (2300 cells/uL, 96% neutrophils), and CSF cultures were negative. Serum ferritin was >15,000 ng/mL, triglycerides were 1234 mg/dL, and soluble IL-2 receptors were 5900 pg/mL. A bone marrow biopsy revealed increased megakaryocytes and hemophagocytosis. An extensive infectious diseases workup was significant for Ehrlichia IgG titers of 1 : 256, and both blood and CSF PCR were positive for *Ehrlichia chaffeensis*. He was continued on doxycycline and treated for HLH with rituximab, dexamethasone, and etoposide. His altered mental status resolved; he was extubated; his acute renal failure resolved; and he was discharged home on day 16.

The literature search yielded 12 publications (Table 1). We added the above four cases seen locally to the other 30 reported cases in the medical literature with HME-associated HLH. Table 1 summarizes the baseline characteristics of patients with HME-induced HLH, their diagnosis, complications, and outcomes.

Two case series included 18 patients [9, 10]. Patients younger than 18 years of age accounted for 41% (14/34) of the cases. Genetic testing was reported in 7 pediatric cases, and none of the patients carried variants known to predispose to HLH.

Otrock et al. did a retrospective review of all their 157 patients with Ehrlichia at Barns-Jewish Hospital in St. Louis, MO, over 10 years. They found that 56/157 were transplant patients; 10 patients (∼6%) met HLH-04 criteria, including only 1 transplant patient. They estimated the true incidence of HLH in HME to be likely higher as their patients were not checked for all HLH criteria [9]. Cabler et al. reported an incidence of HLH in their cohort of HME of 8/49 (16%) at St. Louis Children's Hospital in Missouri over a 16-year period [10].

This study found that 20/34 (59%) of patients were female. All patients had fever, cytopenia, and elevated ferritin. None of the pediatric patients were immunosuppressed, and many of the adults were not, although immunosuppression status was not reported for 9 patients (not transplant). All but one case with available data were treated with doxycycline. The most common additional treatment was the HLH-94 treatment protocol in 8 patients (23.5%).

Complications were common and included shock, meningitis, seizures, respiratory failure, acute kidney injury, and multiorgan failure. One patient had a relapse of Ehrlichia and HLH; another patient had a relapse of HME; and one patient had a relapse of severe thrombocytopenia that responded to doxycycline. Dahm et al. presented 3 cases with HME and myocarditis associated with HLH; 2 of the patients died [8]. The overall case fatality rate was 17.6% (6/34), and 1/8 of the patients who received the HLH-94 protocol died.

## 4. Discussion

The clinical manifestations of HLH result from an immense inflammatory state caused by prolonged and excessive activation of antigen-presenting cells (macrophages and histiocytes) and cytotoxic T cells. This leads to the disruption of the critical regulatory pathways responsible for the normal termination of the immune/inflammatory response [3].

HME-associated HLH is a rare syndrome but is likely underdiagnosed due to the nonspecific clinical and laboratory presentations that are common in this infection, as suggested by Otrock et al. [9]. *Ehrlichia chaffeensis* primarily infects monocytes, one of the cell types implicated in HLH; it can induce an excessive inflammatory response, which can contribute to a septic shock-like picture, thus placing HLH on the differential diagnosis in patients with a potential tick exposure in an endemic area and presenting with a compatible clinical and laboratory finding.

We presented four cases of HME-associated HLH at our institutions, and adding cases from the literature yielded the largest case series on this topic. HME-associated HLH can occur in immunocompetent and immunocompromised pediatric or adult patients. Genetic testing done in pediatric patients did not show an association with a known predisposition to HLH. This review of the literature showed that HME-associated HLH was more common in females, while other studies of secondary HLH showed slightly increased prevalence in males in the adult population but an equal distribution between boys and girls in the pediatric population [17, 18].

It is important to note that all patients had fever, cytopenia, and elevated ferritin. Soluble Interleukin-2 receptor and NK cell activity were not always analyzed in our literature review. The diagnosis of HME and HLH was delayed in many of the described cases in this review. Myocarditis was one of the main manifestations in four patients. Clinicians should therefore be on alert for myocardial involvement and arrhythmias. Most patients received doxycycline, but it was delayed for many. The case fatality rate of 17.6% is lower than that of secondary HLH associated with other conditions [19]. The reported mortality with HLH ranges from 20 to 88% [20]. Mortality associated with Histoplasma-induced HLH was recently reported at 31% [21].

It is possible that the mortality would have been lower if doxycycline was started very early after the presentation. HME-associated HLH relapsed in at least one case; clinicians should be aware of the possible relapse of HLH with or without the relapse of HME. In addition, HME can also relapse without concomitant HLH relapse.

The limited number of patients and the retrospective nature of this review hinder making conclusions about the most effective therapy for this condition. It is important to look for treatable infectious etiologies for HLH; we recommend starting doxycycline as soon as possible in all suspected HME cases. If doxycycline treatment fails to show improvement after 2–3 days, as the response is usually dramatic, the initiation of immunosuppressive therapy such as the HLH-94 protocol [5] is often considered.

## 5. Conclusions

HME is a treatable etiology of secondary HLH and should be kept high in the differential and regularly screened for in the setting of HLH, particularly if practicing within areas where HME is endemic. A timely initiation of doxycycline can potentially decrease mortality. A delay in the diagnosis of HLH may affect outcomes, and a prompt hematology consultation is recommended. Further studies are needed to evaluate the best treatment for this condition and the role of immunosuppressive therapy.

## Figures and Tables

**Table 1:** Characteristics of patients with Ehrlichia-associated HLH.

Study	Pt #	Sex	Age	HLH-2004 criteria	Immunosuppression	Treatment	Complications	Outcome
Hammoud et al.	1	F	4	Fever, splenomegaly, cytopenia, ↑ferritin, ↑sIL2R, ↓Fbg	None	Doxy Dexa	None	Survived
Hammoud et al.	2	F	67	Fever, cytopenia, ↑ferritin, ↑sIL2R, ↓Fbg, splenomegaly	Autoimmune hepatitis-azathioprine	Doxy Dexa	Relapse of severe thrombocytopenia responded to another course of doxy	Survived
Hammoud et al.	3	M	47	Fever, cytopenia, ↑ferritin, ↑sIL2R, ↓Fbg, BM hemoph, low, splenomegaly, ↑TG	None	Doxy Dexa HLH-94 Rituximab	Meningoencephalitis hemodiaysis respiratory failure	Survived
Hammoud et al.	4	F	69	Fever, cytopenia, ↓Fbg, ↑ferritin, ↑sIL2R, BM hemoph, ↑TG	Alcoholism	Doxy, HLH-94	Seizure, myocarditis, severe AKI	Survived
Agudela Higata et al. [6]	5	M	49	Fever, cytopenia, ↑ferritin, ↑sIL2R, ↓Fbg, BM hemoph, low NK, splenomegaly	Psoriatic arthritis -methotrexate -sulfasalazine	Doxy	Bipolaris fungal pneumonia	Survived
Agudela Higata et al.	6	F	62	Fever, cytopenias, ↑ferritin, ↑sIL2R, ↓ BM hemoph	None	Doxy HLH-94	Respiratory failure	Survived
Cheng et al. [7]	7	M	9	Fever, cytopenia, ↑ferritin, ↑sIL2R, ↓Fbg, BM hemoph	None	Doxy HLH-94	Multiorgan failure ECMO	Survived
Dahm et al. [8]	8	M	41	Fever, cytopenia, ↑ferritin, ↑sIL2R, low NK	None	Doxy	Myocarditis	Survived
Dahm et al.	9	F	60	Fever, ↑ferritin, ↑sIL2R, ↓Fbg, low NK, ↑TG	None	Doxy	Myocarditis and acute CHF	Died
Dahm et al.	10	M	68	Fever, cytopenia, ↑ferritin, ↑sIL2R, ↓Fbg, BM hemoph, low NK	None	Doxy Dexa	Myocarditis, VT, acute CHF, multiorgan failure	Died
Otrock et al. [9]	11	F	52	Fever, cytopenia, ↑ferritin, ↑sIL2R, BM hemoph, ↑TG	N/A	N/A	N/A	Survived
Otrock et al.	12	F	47	Fever, cytopenia, ↑ferritin, ↑sIL2R, ↓Fbg, ↑TG	N/A	N/A	N/A	Survived
Otrock et al.	13	F	59	Fever, cytopenia, ↑ferritin, BM hemoph, ↑TG	N/A	N/A	N/A	Survived
Otrock et al.	14	F	16	Fever, cytopenia, ↑ferritin, BM hemoph, ↑TG	N/A	N/A	N/A	Survived
Otrock et al.	15	M	62	Fever, cytopenia, ↑ferritin, splenomegaly, ↑TG	Bilateral lung transplants	N/A	N/A	Survived
Otrock et al.	16	F	9	Fever, cytopenia, ↑ferritin, ↑sIL2R, ↓Fbg, BM hemoph, low NK, ↑TG	N/A	Doxy HLH-94	Recurrent Ehrlichia and HLH	Survived
Otrock et al.	17	F	7	Fever, cytopenia, ↑ferritin, ↑sIL2R, splenomegaly, ↑TG	N/A	N/A	N/A	Survived
Otrock et al.	18	M	77	Fever, cytopenia, ↑ferritin, ↑sIL2R, ↑TG	N/A	N/A	Septic shock	Died
Otrock et al.	19	M	11	Fever, cytopenia, ↑ferritin, ↑sIL2R, ↑TG	N/A	N/A	N/A	Survived
Otrock et al.	20	F	7	Fever, cytopenia, ↑ferritin, ↑sIL2R, ↓Fbg	N/A	N/A	N/A	Survived
Cabler et al. [10]	21	F	8	Fever, cytopenia, ↑ferritin, ↓Fbg, BM hemoph, splenomegaly, ↑TG	None	Doxy	None	Survived
Cabler et al.	22	F	3	Fever, cytopenia, ↑ferritin, ↓Fbg, BM hemoph, low NK, splenomegaly, ↑TG	None	Doxy, HLH-94, IT MTX, HCT	AKI, hemorrhagic cystitis, CMV viremia	Survived
Cabler et al.	23	F	7	Fever, cytopenia, ↑ferritin, ↑sIL2R, ↓Fbg, splenomegaly, ↑TG	None	Doxy, HLH-94	Shock, multisystem organ failure	Died
Cabler et al.	24	F	1.3	Fever, cytopenia, ↑ferritin, low NK, splenomegaly	None	Doxy	Seizure Ehrlichia meningitis	Survived
Cabler et al.	25	M	6	Fever, cytopenia, ↑ferritin, ↓Fbg, BM hemoph, ↑TG	None	Doxy Dexa	Shock	Survived
Cabler et al.	26	F	5	Fever, cytopenia, ↑ferritin, ↑sIL2R, ↓Fbg, ↑TG	None	Doxy	Shock, resp failure, AKI, seizures	Survived
Cabler et al.	27	M	4	Fever, cytopenia, ↑ferritin, ↑sIL2R, ↓Fbg, ↑TG	None	Doxy	Shock, resp failure, AKI, encephalopathy	Survived
Cabler et al.	28	F	6	Fever, cytopenia, ↑ferritin, ↑sIL2R, ↓Fbg, BM hemoph, low NK, splenomegaly, ↑TG	None	Doxy, HLH-94	PRES, relapsed Ehrlichia	Survived
Mitma et al. [11]	29	M	72	Fever, cytopenia, ↑ferritin, ↑sIL2R, ↑TG	MM, HCT	Doxy, MP, tocilizumab	Refractory shock	Died
Kumar et al. [12]	30	M	63	Fever, cytopenia, ↑ferritin, ↓Fbg, splenomegaly, ↑TG	Renal transplant	Doxy, anakinra, MP	None	Survived
Patel et al. [13]	31	M	63	Fever, cytopenia, ↑ferritin, ↑sIL2R, splenomegaly, ↑TG	None	Doxy	Resp failure	Survived
Naqash et al. [14]	32	F	66	Fever, cytopenia, ↑ferritin, ↓Fbg, BM hemoph, low NK, ↑TG	HIV	Etoposide, dexa	Multiorgan failure	Died
Badireddy et al. [15]	33	M	74	Fever, cytopenia, ↑ferritin, BM hemoph, ↑TG	None	Doxy	None	Survived
Kaplan et al. [16]	34	F	41	Fever, cytopenia, ↑ferritin, ↓Fbg, BM hemoph, low NK, ↑TG	None	Prednisone, IVIG, doxy	AKI, meningitis	Survived

Abbreviations. F: female; M: male; sIL2-R: soluble Interleukin-2 receptor; Fbg: fibrinogen; TG: triglyceride; BM hemoph: bone marrow hemophagocytosis; NK: natural killer cell activity; N/A: not available; Doxy: doxycycline; Dexa: dexamethasone; IT MTX: intrathecal methotrexate; AKI: acute kidney injury; HCT: hematopoietic stem cell transplant; resp: respiratory; VT: ventricular tachycardia; CHF: congestive heart failure; PRES: posterior reversible encephalopathy syndrome; MM: multiple myeloma; MP: methylprednisolone; ECMO: extracorporeal membrane oxygenation; RA: rheumatoid arthritis; IVIG: intravenous immunoglobulin.

## Data Availability

All data are included in the submitted text, tables, and referenced articles.
